# “We All Held Our Own”: Job Demands and Resources at Individual, Leader, Group, and Organizational Levels During COVID-19 Outbreak in Health Care. A Multi-Source Qualitative Study

**DOI:** 10.1177/21650799211038499

**Published:** 2021-10-07

**Authors:** Davide Giusino, Marco De Angelis, Greta Mazzetti, Marit Christensen, Siw Tone Innstrand, Ilaria Rita Faiulo, Rita Chiesa

**Affiliations:** 1 University of Bologna; 2 Norwegian University of Science and Technology; 3 Azienda Unità Sanitaria Locale di Bologna

**Keywords:** workplace mental health, health care, IGLO, JD-R, COVID-19

## Abstract

**Background:**

Interventions tackling COVID-19 impact on health care workers’ mental health would benefit from being informed by validated and integrated assessment frameworks. This study aimed to explore the fitness of integrating the Job Demands-Resources (JD-R) model and the Individual-Group-Leader-Organization (IGLO) framework to investigate the pandemic’s impact on health care workers’ mental health.

**Methods:**

Qualitative data were collected via 21 semi-structured interviews with senior and middle managers and four focus groups with employees (doctors, nurses, health care assistants) from three areas (Department of Emergency, Department of Medicine, Research Institute of Neuroscience) of a large health care institution facing the first wave of COVID-19. NVivo deductive content analysis of text data was performed.

**Findings:**

Several COVID-19-related job demands and resources were found at IGLO levels. Individual-level demands included emotional load, while resources included resilience and motivation. Group-level demands included social distancing, while resources included team support and cohesion. Leader-level demands included managers’ workload, while resources included leader support. Organizational-level demands included work reorganization, while resources included mental health initiatives.

**Conclusions/Application to Practice:**

Integrating JD-R and IGLO proved feasible, as job demands and resources could be categorized according to the individual, group, leader, and organization framework. The findings expand previous studies by filling the lack of knowledge on how job demands and resources might unfold at different workplace levels during a pandemic. Results provide unit-level evidence for designing and implementing multilevel interventions to manage health care workers’ mental health during COVID-19 and future pandemics. Our findings offer occupational health practitioners a suitable approach to perform workplace mental health assessment activities.

## Background

COVID-19 has impacted health care workers’ mental health in terms of increased depression, anxiety, and stress (Li et al., 2021; Salari et al., 2020). In Italy, as of July 2021, 136,786 infection cases have been documented among health care workers (Istituto Superiore di Sanità, 2021), who were the most frequently affected occupation by COVID-19 (Istituto Nazionale Assicurazione Infortuni sul Lavoro, 2020). Pandemic infection and witnessing of death and sufferance constitute a serious challenge to health care workers’ mental health (Portoghese et al., 2021). Several studies have shown the impact of COVID-19 on Italian health care workers’ mental health (Babore et al., 2020; Barello et al., 2020; Bettinsoli et al., 2020; Makowiecki et al., 2020; Rossi et al., 2020; Trumello et al., 2020) and found how stressful the pandemic experience has been for these workers due to many risk factors. Health care workers’ mental illness due to COVID-19 has been shown to threaten the quality of health care services and patient safety (Cheng et al., 2020; Teoh et al., 2020). Therefore, mental health interventions among health care workers facing COVID-19 are needed. However, to be effective, interventions have to be informed by an understanding of barriers to both positive mental health and sources of well-being that should be tackled within a given work environment (Christensen et al., 2020). Consistently, workplace mental health assessments should focus on these two dimensions.

The Job Demands-Resources model (JD-R; Bakker & Demerouti, 2018) allows for the detection of workers’ mental health obstacles and facilitators. JD-R perceives the work environment as a potential source of either positive or negative mental health depending on how the work environment is designed, organized, and managed. In this framework, the work environment is composed of job demands and resources influencing workers’ mental health. On one hand, job demands are physical, psychological, social, or organizational aspects of the job, requiring worker’s physical or psychological efforts and being understandable as workers’ mental health risk factors. On the other hand, job resources constitute physical, psychological, social, or organizational aspects of the job that workers can take advantage of and benefit from to counterbalance the physical, cognitive, and emotional costs implied by job demands; they can be understood as workers’ mental health protective factors. Although job demands and resources can impact mental health independently, resources may buffer demands by enabling employee coping. Also, job demands can be hindering demands (i.e., demands that hinder the optimal functioning of the worker) and challenging demands (i.e., demands that stimulate the optimal functioning of the worker; Van den Broeck et al., 2010). In this model, an imbalance between job demands and resources determines distress. Thus, when job demands exceed resources, poor mental health shows up. Recent studies (Chen et al., 2018) have also integrated workers’ personal resources (e.g., optimism, self-efficacy, hope, psychological capital) into JD-R.

Traditionally, job demands and resources have been investigated at the individual level. However, recent literature has argued in favor of addressing multiple workplace levels of analysis and intervention, which may be more effective than addressing one level only (Bakker & Demerouti, 2018; Chen et al., 2018). Accordingly, the Individual-Group-Leader-Organization model (IGLO; Nielsen et al., 2018) could serve to identify job demands and resources. IGLO is an ecological model positing that workplace mental health causes (i.e., where in the workplace mental health may come from) exist at four levels, namely, individual (I), group (G), leader (L), and organization (O). At the individual level, workers’ mental health can be derived from cognitive, affective, and behavioral factors, encompassing personal variables. At the group level, workers’ mental health can relate to colleague support and workgroup climate, entailing team dynamics. The leader level encompasses line managers’ knowledge, skills and abilities, attitudes, behaviors, and support, referring to managers’ characteristics and actions. At the organizational level, Human Resources Management practices and policies, job design, and occupational health services contribute to promoting or hindering workers’ mental health, pointing to how work is designed, managed, and organized. IGLO is a convenient instrument that can be used both for workplace analysis and intervention (Day & Nielsen, 2017). IGLO can guide workplace mental health assessment exercises aiming to identify different sources of workers’ mental health within the work environment. Intervention-wise, IGLO allows a multilevel approach to workplace mental health interventions, which would be implemented at the different levels identified as workers’ mental health sources.

In this study, we adopted an integrated JD-R/IGLO approach to analyze the COVID-19 impact on health care workers’ mental health in an Italian health care institution facing the first COVID-19 pandemic wave. Integration between JD-R and IGLO is achieved by making individual, group, leader, and organizational levels serve as a classificatory framework for job demands and resources. Thus, job demands and resources might be identified at IGLO levels. We argue that this can also function for COVID-19-related risk and protective factors of health care workers’ mental health. Several COVID-19-related job demands and resources have been previously reported to affect health care workers’ mental health. However, studies have not necessarily used such terminology, nor have they appraised variables according to workplace levels. Also, no study on COVID-19 impact on health care workers’ mental health has performed a deductive qualitative analysis based on a strongly intervention-oriented and scientifically well-accredited integrated framework. Mostly cross-sectional quantitative studies have been conducted, with Britt et al.’s (2021) contribution being the only attempt to deploy a JD-R approach to health care workers’ mental health during COVID-19. Also, within a minority of qualitative studies, researchers have mostly adopted inductive, explorative, and descriptive approaches, with the hybrid inductive-abductive analysis by Hennein and Lowe (2020) and Hennein et al. (2021) constituting the closest attempt to ours to investigate health care workers’ mental health during COVID-19 deploying an ecological framework. In this study, we aimed to fill these gaps because (a) quantitative approaches may be limited (De Man et al., 2021) while qualitative analyses might provide deeper insights (Boot & Bosma, 2021) into a disruptive and still partially unknown phenomenon, namely, COVID-19; and (b) an integrated JD-R/IGLO analysis can inform multilevel interventions to promote health care workers’ mental health in a pandemic. Therefore, we conducted a deductive qualitative content analysis (Hsieh & Shannon, 2005) to examine COVID-19-related individual-, group-, leader- and organizational-level job demands and resources. Our aim was to test the fitness of JD-R/IGLO integration; and to explore COVID-19 impact on health care workers’ mental health, lacking theory-based qualitative analyses.

## Methods

We recruited health care workers from three areas (Department of Emergency, Department of Medicine, Research Institute of Neuroscience) of a large Northern Italian health care institution facing COVID-19 first wave using convenience sampling methods. Available participants were invited to participate by the manager of Health and Safety unit at the hospital, via either emails or direct contacts in the workplace. Forty-seven workers volunteered.

Workers were given an informed consent form detailing participation procedure, study contents, data collection purposes, future data dissemination modalities, participants’ rights, and addressable contacts. Participation was voluntary and could be withdrawn at any time without consequences. Trust-based measures were taken by focus group facilitators by informing participants that their information would be kept confidential by the study researchers. They also explained the importance of every participant’s compliance with maintaining privacy and confidentiality about what was discussed in the groups. Researchers declared they would expect to collect various perspectives, so there would be no correct or wrong version. Researchers encouraged participants’ willingness to share opinions by creating a convivial meeting climate. All participants were granted equal opportunity to contribute to the discussion and could decide not to intervene at any time. This study received ethical approval by the Bioethics Committee of the Alma Mater Studiorum—University of Bologna (Prot. n. 0185076) and complied with the Declaration of Helsinki (World Medical Association, 2013).

### Data Collection

For senior and middle managers, we administered online semi-structured individual interviews via a computer-based teleconferencing platform compliant with the General Data Protection Regulation (EU) 2016/679 (GDPR). Twenty-one individual interviews were completed. This flexible strategy was deemed appropriate to managers due to high unpredictability of their work schedules. Validity of this technique is supported by recent literature (Howlett, 2021). In parallel, we held in-person focus groups (Woodyatt et al., 2016) with employees at devoted meeting rooms at participants’ workplaces. Four focus groups were completed, with the number of participants ranging from six to eight per each. All sessions were audio-recorded via secure smartphone software.

To get on the same page about the topic and to make the research protocol following our theoretical model, before each session, all participants were shown a subtitled cartoon video (NTNU Lectures, 2016) providing an easily accessible description of the JD-R model. Also, at the beginning of each session, all participants were given an oral explanation of the IGLO framework. Then, they were encouraged to answer our exploratory questions by keeping in mind the integration between JD-R and IGLO.

One-hour interviews with senior and middle managers were conducted by two trained researchers between September and October 2020, six months after the first COVID-19 outbreak. Example questions from the interview protocol are “Has the impact of COVID-19 varied throughout the pandemic course, as different phases of it, and have its impact been witnessed at your organization?” “Related to the COVID-19 at work, are there any special issues that have influenced your own and others’ mental health?” “Have you made any initiatives in connection to COVID-19 to protect your workers’ mental health?” and “Do you see any special needs regarding mental health among your workers in the light of COVID-19?” Clarifying examples were also provided, namely, social relationships (different ways of working together, sense of isolation, and loneliness), work–life balance (home office, remote working, children at home), change in work tasks (due to digitalization or new roles, increased demands, technostress), worries (family, infection, job insecurity), infection control (social distancing), and personal protection equipment (PPE; e.g., masks, hand sanitizers), leader and co-worker support. Two-hour focus groups with employees were conducted by two trained researchers in September 2020. Participants were asked COVID-19-related questions, after which clarifying examples were provided. The same procedure as the senior and middle managers was followed, and the same questions were asked. Each focus group participant was advised to respect the confidentiality of what was shared by the other members during the focus group.

### Data Analysis

Recorded data were transcribed verbatim with any identifying information anonymized. One researcher cleaned data by formatting raw data files in a common format. Four native Italian speaker researchers performed deductive content analysis via NVivo version 1.3.1 software (Bazeley & Jackson, 2013). The output of the analysis was then directly translated into English by one bilingual native Italian and English-speaking researcher and approved by three other English-only-speaking researchers.

Qualitative content analysis of text data implies the “systematic classification process of coding and identifying themes or patterns” (Hsieh & Shannon, 2005; p. 1278). It provides a flexible and practical technique to investigate human perspectives into matters of health and illness (Hsieh & Shannon, 2005). Particularly, deductive analysis starts from models or frameworks that determine the initial coding scheme or categories, as well as key themes and relationships among them, and are used as a guide for the coding process (Hsieh & Shannon, 2005; Thomas, 2006). The goal is to provide support or to expand existing theories or conceptualizations.

In our study, the JD-R/IGLO integrated theoretical framework provided the initial coding scheme. Thus, content analysis was performed by searching for COVID-19-related job demands and resources at individual, group, leader, and organizational level. If new themes emerged as subcategories to predefined codes, we deployed a more inductive analytical procedure (Braun & Clarke, 2006). No interrater reliability index calculation was deemed necessary as the starting theoretical framework determined agreement on identified codes since early process phases.

## Results

In the Supplementary Table, we classify health care workers’ mental health COVID-19-related job demands and resources at IGLO levels from previous literature. This classification provides a preliminary test for fitness of JD-R/IGLO integration. Participants included 13 males (28%) and 34 females (72%). Eighteen participants were affiliated with the Department of Emergency (38%), 15 with the Department of Medicine (32%), and 14 with the Research Institute of Neuroscience (30%). Eight participants were senior managers (17%), 12 were middle managers (25%), and 27 were employees (58%); among employees, five were doctors (11%), 17 were nurses (36%), and five were health care assistants (11%).

### COVID-19-Related Job Demands

#### Individual level

At the individual level, demands encompassed concern for infected colleagues, fear of death, precariousness of health status, and fear of infecting oneself and his or her family. For instance, one senior manager stated, “At that moment, you’re afraid of death because, every day, from one moment to another, you could go to resuscitation, . . . they’d intubate you and . . . there was fear of death.” Also, one middle manager stated, “Everyone was a little afraid for their families, for their return home.”

Fear of misdiagnosing the novel medical disease was reported by a doctor, as follows:

What’s left from COVID is . . . the fear of not diagnosing it, the fear of erroneously sending a patient home where he/she could enjoy its social life. COVID is a disease that we don’t know about yet, so the biggest fear is sending home a person who maybe has it. . . . And now, in anticipation of . . . seasonal influenza, the overlap between COVID and influenza also becomes an issue.

Exposure to patients’ deaths contributed to workers’ emotional load, especially due to interpersonal distancing safety measures resulting in less humane ways of communicating to patients’ family members. For example, one middle manager indicated that “Death was communicated by telephone . . . this was one of the most stressful events for me personally: Communicating death over the phone was one of the most psychologically taxing things; even now remembering it disturbs me.”

Proximity to family emerged as a need of health care workers facing COVID-19. Relatedly, as physical distancing forced health care workers to greater use of information and communication technologies (ICTs), both with colleagues and their own or patients’ families, some employees reported difficulties in using digital tools so intensively.

#### Group level

At group level, social distancing was reported to hamper both formal and informal team interactions and communications, as it was reported by one middle manager, as follows:

Certainly, one negative aspect of COVID is the distance . . . we’re a close working group, and we like to work close together, we like to collaborate, we like to exchange opinions, and . . . we like to stay . . . close together to talk. Distance works against us because we can’t “huddle together” anymore, so we can’t even take a break. . . . This is a very negative aspect of COVID. . . . This has had a great impact . . ., this crisis of the relational aspect.

Information exchange had been made difficult by social distancing despite ICTs. Mention was made of communication about organizational changes. Besides functional aspects, social distancing was reported to make it harder to keep good team climate. In this regard, one senior manager indicated that

Even just trivially, the meeting. . . . We’re talking about how to keep the climate, how to act discussions. . . . Think of the difficulty we have, as departments, as coordination, in reaching the staff. We used to have the departmental meeting, everyone came, you used to talk and, at least, you managed to do it. Now . . . the meetings are all in small groups, but this means that the coordinator must speak six times because, at each shift change, you take the group off in the morning, on in the afternoon, and you have the meeting. . . . Or, trivially, it was sometimes a moment of pause—no?—to say “come on, it’s the birthday of. . . . We’ll stop for a moment in the kitchen”: It became those ten minutes of breath, of oxygen that, every now and then, are good for you. We can’t do all this anymore.

Social distancing was sometimes reported to make teams’ life even more complicated due to lack of proper workspaces to comply with it.

#### Leader level

At leader level, managers’ workload increased significantly during COVID-19, resulting in higher time pressure and prolonged working hours. One middle manager reported, “My presence was constant during that period—at least 15 hours a day.” Similarly, one senior manager indicated that

I worked every day of the week from morning: I clocked in at 7.30–8am, but at home I had already looked at the mail from 4.30–5am; I came home in the evening at 9am and looked at the mail until midnight or 1am, and I fell asleep on the computer.

Middle managers’ workload was reported to be high as they are the first-instance reference point to employee issues, as well as in between fulfillment of employees’ needs and achievement of organizational goals. This was explained by one middle manager stating that “we’re a truly pivotal role . . . we’re between the management, which sets certain objectives, and the operators . . . —you represent the organization, you represent the management, so, for any problem, they interface with you.” Complementarily, one senior manager reported, “Everyone is looking for the coordinator: . . . for the change of shift . . . and so on. It is a catalyst. . . . I constantly hear middle managers telling me that they are pulled in many directions.”

#### Organizational level

At the organizational level, indicating the magnitude of COVID-19 impact, the pandemic was often framed through a natural disaster metaphor, as a “tsunami” or a “cataclysm.” Relatedly, work reorganization was the most cited demand. COVID-19 outbreak was reported to impose logistical changes to organizational structures and hospital wards, as well as changes to types of provided care and assisted patients, which had to be implemented rapidly. In this regard, one health care assistant reported that “There were continuous updates during the emergency.” One middle manager reported an exemplifying experience, as follows:

from Friday afternoon to Saturday morning, the ward was completely empty of normal patients and filled with these other patients—which was completely different because, before, they were all elderly . . . people, many of them pathological; and, from evening to morning— . . . in 24 hours—the ward was filled with young people with a respiratory disease, with a current swab that you didn’t know if it was positive or not. Many . . . tested . . . positive. So, the target group of patients had really changed.

Relatedly, another health care assistant expressed that more instructions about the continuous organizational changes would have supported workers’ feeling of control over the situation, as follows: “the management of updates during the emergency: Some more positive feedback about who was managing the procedures correctly perhaps would have helped the group understand what the right direction was.”

Cooperative work reorganization processes were accomplished by exploiting web-based ICTs. Modifications were reported about executing daily work activities due to having to adopt contagion prevention measures. For instance, one middle manager reported that

we got used to communicating by telephone, by videocalls, while we were used . . . to having an open intensive care unit where relatives would come in, stay inside with us, work with us together with our patients, and we’d communicate with them in a built environment, a small living room . . . During the pandemic—imagine that!

Also, one senior manager stated, “the COVID experience . . . certainly put a strain on everyone because it totally changed the way we worked: Even just working the whole shift with the PPE.”

Both the forced work reorganizations and COVID-19 outbreak itself resulted in increased workload, higher time pressure, and prolonged working hours. This was the case for both managers and employees. For example, one middle manager said, “from 30 operators, I found myself managing 50.” Similarly, one nurse stated, “During the COVID period, we gave 100% availability by giving up our holidays, doing double shifts.”

This theme linked to a sense of unpreparedness, encompassing emotional surprise toward the unprecedented pandemic phenomenon and the fast changes in work practices. This was condensed in the words of one middle manager below:

Now, we’ve a . . . COVID ward, with very young nurses inside who find it very difficult to deal with a patient who’s so critical, complex, in a situation that’s complex regardless, because you work in clothes, . . . in a new environment that you don’t know, where nobody has told you what . . . to do or what is normally done.

Prolonged work shifts determined by the emergency were reported to negatively affect work–life balance, for instance, by one middle manager who indicated that “It has a . . . negative impact because it affects family management . . . almost all of them have a family life, so children, and so on. . . . Especially in the lockdown, they suffered a lot from this shift.”

From a different perspective, one senior manager appraised COVID-19-related work reorganizations as a learning opportunity that the health care institution should exploit to develop more flexible organizational models to manage future difficulties.

### COVID-19-Related Job Resources

#### Individual level

At individual level, personal resources were mentioned, namely, proactivity, flexibility, adaptability, engagement, resilience, extra-role behaviors, motivation, personal initiative, and enthusiasm. Particularly, one middle manager talked about motivation, as follows: “at that moment, each of us brought out our personal drive, our personal motivation to always want to do better, even in difficult times . . . we went ahead despite the difficulties.”

Another middle manager especially referred to adaptability, as follows:

So, what I noticed immediately was the great openness and willingness of colleagues to do things that clearly were not done before. So, this is . . . a different mental approach that the doctors had to put together. And I’ve seen that, on this occasion. They’ve been very helpful . . . this was my first impression, even looking back on it afterwards: It was the great helpfulness of everyone. . . . I didn’t see anyone backing out . . ., I didn’t see anyone taking a back seat. They also worked many more hours than normal . . . we were a bit self-taught. . . . And there had to be a change of mentality and rapid adaptation to the new situation. . . . I noticed that there was a good adaptation to the situation.

Another middle manager especially referred to resilience, as follows: “we were resilient because nobody gave up. . . . we all held our own.”

These findings suggest that COVID-19 impact on health care workers should not necessarily be framed negatively, as it was also argued by one senior manager as follows:

COVID . . . gave an enormous stimulus; they all did a lot, because it was a very strong situation, but it had the effect of . . . motivating them . . . from this point of view, it was very positive . . . there was a great deal of participation: People who travelled many kilometers to come and give us a hand, a great deal of willingness to welcome people who, coming from other hospitals, didn’t know what the minimal organization was like. So, it was very tiring, but exciting.

#### Group level

At group level, most resources were reported, namely, mutual support, increased cohesion, solidarity, teamwork, and inter-professional cooperation. One nurse referred to group-level resources as follows: “We all started from the same base, and this created wonderful groups, because there was daily talking and discussion.”

A shared perception was expressed about COVID-19’s positive effect on interpersonal relationships within workgroups. Feeling “all in the same boat” facilitated dealing with the pandemic situation. This emerged, for instance, from the following words of one middle manager:

a marvelous atmosphere was created that I’d never have thought possible . . . and a kind of solidarity and extremely positive atmosphere was created despite the heavy workload . . . because we felt very close to each other . . . the . . . infectious disease . . . meant that this new group immediately came together . . . with COVID, a great deal of solidarity was created, . . . there was a coexistence of a couple of months where everyone appreciated each other, was well integrated.

A similar report was provided by another middle manager, as follows:

certainly, working closely with everyone made this period less burdensome. . . . It wasn’t easy, but the key element that led us, however, to make stress a source of wellbeing, was the collaboration. . . . Our working together has made us feel less alone, that’s for sure. . . . The team collaboration . . . made everything flow spontaneously.

The pandemic situation was reported to have transformed previous inter-professional conflicts into a collaborative, cohesive, and mutually supportive climate. One middle manager reported that

the way of looking at each other has changed . . .: There is a mutual esteem that there was not before; so, one has seen you working there, you have seen him working there, you have seen the shifts—and you look at them in a different way. And that’s nice.

Another middle manager reported, “we all worked and there was no longer the professor, the first-level manager, the nurse.”

Nonetheless, employees reported the above group aspects were already forgotten after the first COVID-19 wave, but that improvements at group level were needed anyway. As the groups already started to feel less cohesive and collaborative, participants stated that the COVID-19 experience should teach everybody much about teamwork.

#### Leader level

At leader level, leaders’ support was reflected by managers’ prolonged availability, motivating behaviors and role modeling, despite sometimes leaders feeling unprepared as much as employees to deal with the unprecedented situation. For example, one middle manager said that

I felt, . . . as a manager, . . . as a nurse, . . . as a human being, that I should not leave everything at the mercy of the wave. My presence was constant. I was, above all, a psychological support . . . whereas I, first, was disoriented because I didn’t know what to do. However, I always said, “Let’s do it, let’s see together and let’s face this thing.”

Supportive leadership was also deployed by recognizing their own inability to manage certain situations and addressing employees as competent professionals, for instance, advising them to consult psychologists who had been activated specifically. Here, leader’s awareness of available organizational services would arguably make the difference. Furthermore, professional gratification was reported by one senior manager as instrumental to provide support, as follows: “I wrote an e-mail where I told them that ‘I am particularly proud of your ability’—I had sent it to everyone . . . —like that, even fast, right?”

Leader support was also shown through granting employees all necessary PPE to survive the exceptional work situation. Scheduling team meetings to verbalize feelings related to facing the pandemic was reported by one senior manager as a good practice to offer leader support, as follows:

immediately following the COVID explosion, . . . I organized a meeting with them . . . and I said, “Look, I have nothing to communicate to you: I just want to listen to you. . . . Take out everything you have accumulated in COVID” . . . . And it was very much appreciated.

#### Organizational level

At organizational level, some workers perceived a resource in mental health initiatives and the availability of different organizational well-being services that the health care institution implemented to mitigate COVID-19 psychological impact. In this regard, one nurse reported that “With the COVID emergency, there was the possibility of meetings with consultants, both external and internal to the organization, at the request of the individual concerned.” Similarly, another nurse said, “A psychological support service was included during COVID.”

However, this finding was not univocal, as other interviewees reported opposite accounts, as was the case for another nurse, stating that

at the end of the big stress related to COVID, it would have been nice to ask, on a structured level, “how are you doing?”; but there wasn’t that moment where we . . . see how to deal with it.

Support from ICTs to safely perform daily work activities was also reported as an organizational-level resource, related to how work was managed during the outbreak, for example, by one middle manager as follows: “our only source of communication with patients’ families were videocalls.”

PPE provision was reported by one middle manager to ensure health care workers’ safety, both physically and psychologically, as follows:

I’d say that, if a phase 2 or 3 ever happens, it’ll never be the same as before. We’re all much better prepared. Quite simply, we’ve the equipment. The company’s stockpiled. . . . I see this from colleagues who’ve a slightly more relaxed attitude towards the use of devices.

## Discussion

Based on a JD-R/IGLO analytical framework, we performed a deductive content analysis on COVID-19 impact on health care workers’ mental health. Job demands and resources were found at individual, group, leader, and organizational level, as shown in Table 1. At individual level, emotional load aligns with recent findings about influence of pandemic fear of death and exposure to sufferance (Portoghese et al., 2021) on health care workers’ mental health. Our study suggests additional concerns to consider, namely, those for infected colleagues, families, and misdiagnosing. Yet, health care workers deployed personal resources to face the challenging situation, which is similarly reported elsewhere. Furthermore, findings about employees exploiting ICTs to seek family proximity seems consistent with Luo et al. (2020) reporting that “staff experienced psychological stress or emotional changes . . . caused by family health and disease related issues. Most of them managed their emotions by self-control and video calls with their families” (p. 1).

**Table 1.** Findings of Health Care Workers’ Mental Health COVID-19-Related Job Demands and Job Resources at Individual, Group, Leader, and Organizational Levels

	COVID-19 demands	COVID-19 resources
*Individual*	Emotional load (concern for infected colleagues, fear of death, fear of infecting oneself and own family, fear of misdiagnosing, exposure to death), Need for family proximity, Digital illiteracy	Proactivity, Flexibility, Adaptability, Engagement, Resilience, Extra-role behaviors, Motivation, Personal Initiative, Enthusiasm
*Group*	Social distancing hampering interactions, communications, information exchange, and social climate	Mutual support, Increased cohesion, Solidarity, Teamwork, Inter-professional cooperation
*Leader*	Leaders’ increased workload	Leaders’ support
*Organization*	Increased workload, high time pressure, prolonged working hours, Work–life conflict, Work reorganization	Mental health initiatives, Information and communication technologies, Personal protective equipment

*Note.* COVID-19 = coronavirus disease 2019.

At group level, working for a common good was associated with motivation and lowered inter-professional conflict. Arguably, perceiving a common “enemy” and the shared goal of fighting it determined social recategorizations whereby ingroup and outgroup have come to feel as one. This seems consistent with Makowiecki et al. (2020) stating that “war is happiness, in the sense that increased trust, friendship and collaboration in the fight” (pp. 35–36). Notably, only managers reported issues about social distancing and teamwork, which may indicate leaders’ sensitivity toward the health status of teams they are responsible for.

At leader level, leaders’ workload appeared most relevant. Leaders’ behaviors are crucial for ensuring workers’ mental health (Day & Nielsen, 2017; Nielsen et al., 2018). However, high workload may prevent leaders from behaving in ways facilitating employees’ mental health. Besides, leader support was reported as a resource, also manifesting in informing employees about adequate mental health services to consult. However, this was reported as a reactive behavior to employees raising psychologically relevant issues, rather than as a proactive behavior aiming to prevent employees’ mental illness.

At organizational level, sudden work reorganization was reported as a major demand. COVID-19 has determined logistical changes to organizational structures and hospital wards, as well as modifications to work practices, types of provided care and assisted patients, resulting in a sense of unpreparedness toward the unprecedented pandemic phenomenon. In contrast, COVID-19-related mental health initiatives were reported as resources. However, a discrepancy could be registered between perceived lack of initiatives availability and their actual availability (e.g., individual coaching, provision of psychological support services both in-presence and by phone, self-help groups), even within the same organizational areas. This may suggest that communication about organizational initiatives might have been improved. Also, this lack of communication might link to the aforementioned leaders’ reactivity instead of proactivity in informing employees about adequate mental health services; that is, leaders may be expected to act as communicative bridges between the organization and employees because, if they do not do so, initiatives do not get known by potential beneficiaries. Nevertheless, we underline how difficult it can be to distribute attention to all communications during a health emergency.

We conducted our study involving both managers and employees. However, no relevant differences between managers and employees emerged from our qualitative analysis in terms of types of reported job demands and resources nor in terms of number of reported demands versus number of reported resources. It only seems that, compared with managers, employees reported more demands at the organizational level of analysis.

This study has limitations. Although adopting the JD-R/IGLO analytical framework should have provided researchers with a shared mental model ensuring consistency of findings, these derive from an interpretive process, which is inherent to qualitative research and might be biased toward adopted theories (Hsieh & Shannon, 2005). Deductive coding might mitigate subjectivity-related inconsistencies across researchers, which more likely occur in inductive coding where no initial framework is adopted. Also, multiple data sources (i.e., managers and employees from three hospital areas) might enhance the credibility of our analysis (Hsieh & Shannon, 2005); in fact, the wide variety within our sample is why we define our study as “multi-source.” Recall and self-report bias (Stone et al., 2002) might have occurred, and generalizability remains questionable. Boundaries between IGLO levels were sometimes blurred; for instance, differences between individual and leader level were not always straightforward, as leaders are individuals themselves.

Overall, JD-R/IGLO integration proved feasible. Demands and resources could be categorized according to the individual, group, leader, and organization framework. This couples two separate workplace mental health research strands, which are linked to relevant multilevel interventions for health care institutions. IGLO offers a framework to classify demands and resources based on their source, whether they are inherent in individuals, social contexts, or ways work is organized, designed, and managed. Therefore, we provide an integrated model, which can be used in future workers’ mental health studies.

## Implications for Occupational Health Practice

First, we offer occupational health practitioners a suitable approach to workplace mental health assessment activities. As we tested in the present study, conducting similar exercises based on a JD-R/IGLO integrated framework, which means looking for both job demands and job resources simultaneously at individual, group, leader, and organizational level, may prove fruitful and informative. In our study, qualitative methods proved effective to capture both risk and protective factors of mental health in the given work environment; however, quantitative methods may not be discarded a priori as long as they rely on a JD-R/IGLO integrated framework.

Second, multilevel interventions to reduce demands and enhance resources in the working environment can be derived from JD-R/IGLO-based workplace mental health assessment activities. For instance, positive stress management, mindfulness, job crafting, and other techniques generally addressing personal resources (Gilbert et al., 2017) might be implemented at the individual level. Team coaching (Clutterbuck, 2010) may be carried out at group level. Managerial or positive leadership development training (Peláez et al., 2020) might be implemented at the leader level. Finally, job redesign interventions (Holman & Axtell, 2016) may be implemented at the organizational level. So, practical implications are also held for design of mental health interventions in health care organizations, and for management of health care workers’ mental health during next COVID-19 waves and future pandemic outbreaks. Our study suggests that both managers and employees would benefit on multilevel interventions as they seem to experience the same type of challenges and opportunities during a crisis like COVID-19.

**Applying Research to Occupational Health Practice**

Several COVID-19-related job demands and job resources were found at individual, group, leader, and organizational level throughout a multi-source, interview, and focus group-based deductive qualitative content analysis of the first pandemic wave impact on health care workers’ mental health at a large Northern Italian health care institution. Demands included emotional load, need for family proximity, and digital illiteracy (individual level); social distancing (group level); increased workload (leader level); time pressure, prolonged working hours, work–life conflict, and work reorganization (organizational level). Resources included adaptability, resilience, and personal initiative (individual level); support, cohesion, solidarity, teamwork, and inter-professional cooperation (group level); managerial support (leader level); mental health initiatives, ICTs, and PPE (organizational level). Occupational mental health practitioners can use the retrieved knowledge to design multilevel interventions to promote health care workers’ mental health during pandemics. The proposed JD-R/IGLO integrated analytical framework can be deployed to perform workplace mental health assessment activities.

## Supplemental Material

sj-docx-1-whs-10.1177_21650799211038499 – Supplemental material for “We All Held Our Own”: Job Demands and Resources at Individual, Leader, Group, and Organizational Levels During COVID-19 Outbreak in Health Care. A Multi-Source Qualitative Study Click here for additional data file. Supplemental material, sj-docx-1-whs-10.1177_21650799211038499 for “We All Held Our Own”: Job Demands and Resources at Individual, Leader, Group, and Organizational Levels During COVID-19 Outbreak in Health Care. A Multi-Source Qualitative Study by Davide Giusino, Marco De Angelis, Greta Mazzetti, Marit Christensen, Siw Tone Innstrand, Ilaria Rita Faiulo and Rita Chiesa in Workplace Health & Safety
