# Supplementary material for: “We All Held Our Own”: Job Demands and Resources at Individual, Leader, Group, and Organizational Levels During COVID-19 Outbreak in Health Care. A Multi-Source Qualitative Study
Source: Workplace Health Saf. 2021 Oct 7;70(1):6–16. doi: 10.1177/21650799211038499 (PMC8503930; doi:10.1177/21650799211038499)
Supplement: sj-docx-1-whs-10.1177_21650799211038499 – Supplemental material for “We All Held Our Own”: Job Demands and Resources at Individual, Leader, Group, and Organizational Levels During COVID-19 Outbreak in Health Care. A Multi-Source Qualitative Study [file sj-docx-1-whs-10.1177_21650799211038499.docx]

**Supplementary Table.** Healthcare workers’ mental health COVID-19-related job demands and job resources at individual, group, leader, and organizational levels from previous literature.

|  | *COVID-19 Demands* | *COVID-19 Resources* | *References* |
| --- | --- | --- | --- |
| *Individual* | Death and dying, Worrying getting and transmit virus, Uncertainty, Avoidance strategies, Emotional demands, Mental demands, Fatigue, Feeling of isolation and discrimination, Worries about job and economy, Negative metacognitions | Positive work attitude, Meaningful work, Resilience (expressive flexibility, context sensitivity), Self-efficacy, Coping strategies (humor, refocusing on planning, positive reappraisal), Professional identification and commitment, Recovery experiences, Self-care, Personal fulfilment, Hardiness | Abbas et al., 2021; Babore et al., 2020; Barello et al., 2020; Bettinsoli et al., 2020; Bozdag & Ergün, 2020; Canestrari et al., 2021; Correia & Almeida, 2020; Cui et al., 2020; Delgado-Gallegos et al., 2020; Di Monte et al., 2020; Fauzi et al., 2020; Finell & Vainio, 2020; Hennein & Lowe, 2020; Hou et al., 2020; Hu et al., 2020; Huang et al., 2020; Johnson et al., 2020; Lai et al., 2020; Lenzo et al., 2021; Luceño-Moreno et al., 2020; Maiorano et al., 2020; Man et al., 2020; Mekonen et al., 2021; Ou et al., 2021; Ramaci et al., 2020; Sadati et al., 2020; Seçer et al., 2020; Teshome et al., 2020; Tiete et al., 2021 |
| *Group* | Social stigma, discrimination experience, workplace violence | Health climate (workgroup), Social support, Work community, Teamwork, Team communication | Abbas et al., 2021; Cui et al., 2020; Finell & Vainio, 2020; Hennein & Lowe, 2020; Hou et al., 2020; Ramaci et al., 2020; Rojas-Ocaña et al., 2020; Sadati et al., 2020; Yang et al., 2021 |
| *Leader* |  | Health climate (supervisor) | Abbas et al., 2021 |
| *Organization* | Inadequate preparation, Heavy workload, Work-family unbalance, Changes in working patterns, New ways of working, Front-line work, Lack of personal protective equipment, Role conflict | Health climate (organization), Adequate training, Organizational procedural justice, Personal protective equipment, Management support, Hospital resources and grade | Abbas et al., 2021; Barello et al., 2020; Correia & Almeida, 2020; Cui et al., 2020; Hennein & Lowe, 2020; Huang et al., 2020; Ide et al., 2021; López-Cabarcos et al., 2020; Luceño-Moreno et al., 2020; Luo et al., 2020; Maiorano et al., 2020; Mekonen et al., 2021; Mo et al., 2020; Morgantini et al., 2020; Rossi et al., 2020; Tiete et al., 2021; Trumello et al., 2020; Yang et al., 2021; Zhang et al., 2020 |

*Note.* Cross-sectional quantitative studies are Abbas et al. (2021), Bozdag & Ergün (2020), Correia & Almeida (2020), Delgado-Gallegos et al. (2020), Fauzi et al. (2020), Finell & Vainio (2020), Hou et al. (2020), Hu et al. (2020), Huang et al. (2020), Ide et al. (2021), Johnson et al. (2020), Lai et al. (2020), López-Cabarcos et al. (2020), Luceño-Moreno et al. (2020), Luo et al. (2020), Man et al. (2020), Mekonen et al. (2021), Mo et al. (2020), Morgantini et al. (2020), Ou et al. (2021), Seçer et al. (2020), Teshome et al. (2020), Tiete et al. (2021), Zhang et al. (2020). Qualitative studies are Alizadeh et al. (2020), Arnetz et al. (2020), Catania et al. (2020), Cui et al. (2020), Hennein & Lowe (2020), Liu et al. (2020), Rojas-Ocaña et al. (2020), Sadati et al. (2020), Sun et al. (2020).
